# The change in attitude and knowledge of health care personnel and general population following trainings provided during integration of mental health in Primary Health Care in Iran: a systematic review

**DOI:** 10.1186/1752-4458-3-15

**Published:** 2009-06-25

**Authors:** Naghmeh Mansouri, Banafsheh Gharaee, Seyed Vahid Shariat, Jafar Bolhari, Reza Yousefi Nooraie, Afarin Rahimi-Movaghar, Narges Alirezaie

**Affiliations:** 1 Mental Health Research Center and Tehran Psychiatric Institute, Iran University of Medical Sciences, Tehran, Iran; 2 Department of Psychology, Tehran Psychiatric Institute and Mental Health Research Center, Iran University of Medical Sciences, Tehran, Iran; 3 Department of Psychiatry, Tehran Psychiatric Institute and Mental Health Research Center, Iran University of Medical Sciences, Tehran, Iran; 4 Center for Academic and Health Policies, Tehran University of Medical Sciences, Tehran, Iran; 5 Iranian Research Center for HIV/AIDS (IRCHA), Tehran University of Medical Sciences, Tehran, Iran; 6 World Health Organization Collaborating Center, Tehran Psychiatric Institute, Iran University of Medical Sciences, Tehran, Iran

## Abstract

**Background:**

Mental health has been integrated in the primary health care program in small cities and villages of Iran in a national level since the late 1980s. We performed a systematic review of literature to investigate the effect of education on change in attitude and knowledge of mental health care providers and the population covered in the program during the recent two decades in Iran.

**Methods:**

Electronic bibliographic databases including Pubmed, PsycINFO and EMBase as well as the main Iranian databases (Scientific Information Database, IranMedex, IranPsych, and IranDoc) were searched. Additionally, hand searching, personal contacts and tracking of reference lists were performed. All of the studies which compared the attitude and knowledge of the related population before and after an educational intervention were recruited.

**Results:**

Six articles met the inclusion criteria and entered the review. All of these studies showed an improvement in the attitude and knowledge of the studied population. Although the studies were different in many respects, a meta-analysis on the two more similar studies showed a significant effect of training on long term improvement of the knowledge and attitude of the population.

**Conclusion:**

A short term training improved knowledge and attitude of the population and health personnel immediately after the intervention. There is also evidence for a long term change in the attitude and knowledge of general population after short term training.

## Background

Integrating mental health services into Primary Health Care (PHC) is one of the most fundamental strategies expected to provide several advantages for the service consumers [1]. These advantages include reduced stigma, improved access to care, reduced chronicity and improved social integration. In addition, such integration provides services more compatible with better protection of human rights. Better overall health outcome of the consumers and improvement of human resources capacity are among other benefits of such integrated systems [2].

Iran with a population of 70495782 is located in the Middle East and has a literacy rate of 84% [3]. Seventy one percent of the population is living in the cities and 29% form the rural population. The PHC system has greatly expanded in Iran since 1984 [4]. The core component of the PHC lies in its referral-based district units, with the most peripheral level (Health House) run by a native health worker, called *Behvarz*. They generally pass a minimum of five years of formal education and a two year special intensive training on public health. These health workers are assumed to serve around 1500 residents following a strictly defined guideline. A wide range of family health services including immunization, case finding and follow-up are provided by *behvarz*es. They also present family planning, health education, and environmental health services for their covered population. Every Health House is supervised by second level "Rural Health Centers" covering around a population of 9,000. A team of skilled health technicians provide diagnosis and outpatient care services for referral cases under the supervision of a general physician. The official equivalent of Rural Health Center in the cities is implemented in Urban Health Center. These centers perform the same type of functions for a population of 12,000. Urban areas lack grass root facilities like Health Houses, although there have been some efforts for developing such a system through recruiting local volunteers, called *Rabet*. The third and fourth levels provide more specialized services.

The program for integration of mental health in PHC in Iran began in late 1980s and now it is one of the few programs in the world that have been implemented in a nationwide scale [5]. The program has a set of core activities, which include:

i. Mental health education to the community, families and schools

ii. Active identification and referral of patients with major mental health problems, including psychosis, epilepsy, drug dependence, and mental retardation; as well as follow-up, home visit, and preparation for the reintegration of the patients into the community

iii. Implementation of a data registry system

Knowledge and attitude of the community and health care workers are important factors that could play a role in successful implementation of the program. It has been shown that adequate knowledge and positive attitude of the population towards patients with mental disorders could help to reduce the related stigma [6]. In addition, health care workers could better detect mentally disordered patients if they bear a positive attitude toward them [5]; otherwise, problems may arise in implementation of the program [7].

Some studies have showed that the knowledge and attitude of both the community and the health care workers changes immediately and significantly, some time after the training program [8].

Moreover, WHO performed a collaborative study in the 1980s to evaluate these outcomes in seven countries, with the same design. These studies have shown a positive effect on the attitude and knowledge of the community and personnel 18 to 24 months after the intervention [9]. However, the design and implementation of the integration program have not been the same across countries; therefore, any attempt to perform a secondary data analysis should be performed either in a single country or in countries with similar programs. As previously mentioned, the integration program in Iran has been performed in a national scale, and it therefore covers a variety of populations with different characteristics.

As a result, Iran provides a potentially unique field for the studies of the integration program, and the meta-analysis of these studies, if applicable, could be especially informative. To our knowledge, this is the first systematic review on the change in attitude and knowledge of health personnel and covered population in the program of integration of mental health in PHC after an educational intervention. We systematically reviewed the available evidence of the recent two decades to answer to this question. Case finding is also one of the main objectives of the integration program that has been addressed in another parallel systematic review [10].

## Methods

The initial searches were performed in November 2006. A repeated search was performed in September 2008 to update the data.

### Search strategy

The electronic bibliographic databases including PubMed, PsycINFO, and EMBase and available Iranian electronic databases including IranPsych, Scientific Information Database, IranMedex, and IranDoc were searched [see Additional file 1]. Additionally, the non-electronic documents of the IranPsych (including the conference proceedings and thesis and dissertation abstracts) as well as the related documents of the WHO Collaboration Center in Tehran Psychiatric Institute and the Bureau of Psychosocial Health and Addiction of the Ministry of Health and Medical Education, were hand searched. Reference lists of the selected articles were also scanned for relevant resources.

Studies with the following criteria were included:

1. Participants were selected either from general population in the areas where the integration program was running or from at least one of the health personnel subgroups working in the program including *Behvarz *and *Rabet *(Health workers of the first level of PHC in rural and urban areas, respectively), health technicians and general practitioners working in the second level.

2. An educational intervention (as a part of integration program) was implemented to improve the knowledge or change the attitude of the studied population.

3. Subjects were assessed both before and after the intervention with or without concurrent comparison with a comparable group from an area not covered by the integration program.

4. At least one of the measures including level of knowledge or attitude of the subjects was assessed.

Twenty one studies that did not meet these criteria were excluded. The excluded studies and the reason for their exclusion are as follow: A sample of two studies was not from areas covered by PHC [11,12], seven studies were reviews and lacked primary data [13-19], three studies did not compare the variables before and after the training [20-24], three studies were reduplications (those parts of the findings that were relevant to our review were also reported in other articles, but they included non-reduplicated data too) [25-27], two studies focused only on attitude toward epileptic patients [28,29], one study assessed the knowledge about non-mental disorders [30], and one study did not present the necessary details of the findings in a way that could not be used in the review [31]. Three researchers assessed the abstracts and decided which of them should be selected for full text evaluation. Then a checklist was used to evaluate the quality of the retrieved full texts in terms of the objectives, inclusion and exclusion criteria, variable definitions, sampling method, and validation of the employed tests.

### Data extraction

The following data were extracted from each study: sampling method and characteristics of the sample and comparison group, studied variables, details of intervention, characteristics of assessment questionnaires, including the type and range of scoring, and mean and standard deviation of the scores before and after the intervention.

### Description of studies

**Figure 1:**
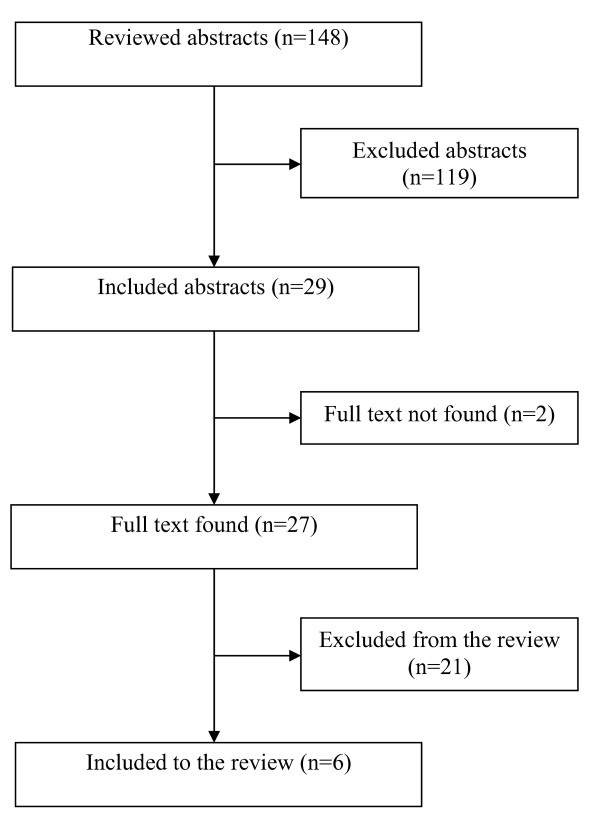
**Flowchart for selection of the studies for the review**.

Twenty nine abstracts were selected for further evaluation of their full text, among which 6 studies met the inclusion criteria of the review (figure 1). We could not access the full text of two studies and the other twenty one studies were excluded because they did not meet the inclusion criteria. The excluded studies and the reason for their exclusion are as follows: a sample of two studies was not from areas covered by PHC [11,12], seven studies were reviews and lacked primary data [13-19], five studies did not compare the variables before and after the training [20-24], three studies were reduplications (those parts of the findings that were relevant to our review were also reported in other articles, but they included non-reduplicated data too [25,26], two studies focused only on attitude toward epileptic patients [28,29], one study assessed the knowledge about non-mental disorders [30], and one study did not present the necessary details of the findings in a way that could not be used in the review [31].

Three researchers assessed the abstracts and decided which of them should be selected for full text evaluation. Then a checklist was used to evaluate the quality of the retrieved full texts in terms of the objectives, inclusion and exclusion criteria, variable definitions, sampling method, and validation of the employed tests.

A summary of the characteristics of included studies is presented in table S1 [see Additional file 2]. Some of the studies were performed on more than a single subgroup of subjects and/or used different assessment methods for the sample subgroups; therefore, the total sum of the studies in the text or tables is more than six.

Studies differed in the ways they assessed the attitude and knowledge of the samples. Three studies reported a single composite score for the two variables, but others evaluated them as separate variables and reported either a quantitative (mean and standard deviation in three studies) or qualitative (percent of the subjects having the variable in one study, i.e. having adequate knowledge and a positive attitude) measure for either variable (tables 1, 2, and 3). The samples in the reviewed studies included *Behvarz *(four studies), general population (four studies), and other subgroups (one study). Most of the studies did not describe validity and reliability of the utilized instrument. In addition, none of them explained the inclusion and exclusion criteria.

**Table 1:** Studies reporting mean and SD of the knowledge and attitude of the subjects as a single variable before and after the training

Author	Sample	Attitude & Knowledge			
		
		Scores range	Pre (M, SD)	Post (M, SD)	Statistical sig.
Shahmohamadi, 1991	*Behvarz:*				
	(Experimental)	0–50	22.96 ± 47.73	40.81 ± 11.08	t = 11.65*
	(Control)		24.87 ± 21.66	28.64 ± 18.9	p < 0.01
Bolhari, 1995	*Behvarz:*				
	(Experimental)	0–29	17.68 ± 2.84	21.09 ± 2.37	t = 5.63*
	(Control)		16.4 ± 2.78	17.65 ± 2.03	p < 0.01
Kadivar, 1997	*Karshenas*	0–20	6.2 ± 2.9	12 ± 5.1	p < 0.05**
	*Kardan*	0–20	6.2 ± 2.9	12.6 ± 4.1	
	*Behvarz*	0–20	3.6 ± 2.7	10.8 ± 3.7	
	*Behdashtyar*	0–20	5.3 ± 2.4	13.6 ± 3.4	

* This comparison has been made between the attitude scores of the experimental and control group after the intervention, while the pretest scores have not been significantly different

* * The amount of t was not reported in the original document

**Table 2:** Studies reporting mean and SD of the knowledge and attitude of the subjects as separate variables before and after the training

Study		Knowledge				Attitude			
Author	Sample	Before M (SD)	After M (SD)	Score Range	Statistical sig.	Before M (SD)	After M (SD)	Score Range	Statistical sig.

Motallebi, 1996	*Behvarz*	15.43(5.04)	32.52(2.84)	0–38	t = 30.99 p < 0.01	8.39(2.23)	12.58(1.41)	0–26	t = 16 p < 0.01
Davasaz, 2004	General population								
	Experimental	36.36(8.4)	46.9(4.78)	0–58	F = 107.26	6.29(5.7)	14.51(3.36)	0-22	F = 56.43
	Control 1*	32.73(8.85)	35.96(5.44)	0–51	df = 2,270	6.36(4.04)	6.65(3.67)		df = 2,264
	Control 2*	32.04(6.68)	30.94(4.44)	0–42	p < 0.0001	4.43(3.01)	5.20(2.79)		P < 0.0001
Bolhari, 1995	General population								
	Experimental	-	-	-	-	14.33(2.87)	14.7(3.07)	0-22	t = 2.21** p < 0.05
	Control					14.44(2.91)	14(3.04)		
Shahmohammadi, 1990	General population								
	Experimental	-	-	-	-	18.79(22.27)	23.12(24.34)	0–30	t = 9.44** p < 0.01
	Control					18.25(22.75)	20.18(28.75)		

* Control 1 has been selected from the same village and control 2 from two other villages

** This comparison has been made between the attitude scores of the experimental and control group after the intervention, while the pretest scores have not been significantly different

**Table 3:** Studies reporting separate percentages for the knowledge and attitude levels of the subjects before and after the training

Study		Knowledge (Percent)				Attitude (Percent)			
Author	Sample	Pre		Post		Pre		Post	

Bagheri Yazdi, 2002	Trained group (rural)	57	(43)	32	(68)	31	(69)	13.5	86.5
	Control group (rural)	58	(42)	57	(43)	32	(68)	31.6	(68.4)
	Trained group (urban)	52.2	(47.8)	27.5	(72.5)	30.2	(69.8)	15.5	(84.5)
	Control group (urban)	54.4	(45.6)	54	(46)	32	(68)	29.9	(70.1)

Extracted data were entered to RevMan 4.2 Software for further analysis. Because of the fact that the methods and time intervals between training and evaluation were not the same in most studies, we could only perform meta-analysis on two studies.

## Results

We divided the included studies to the three following subgroups based on the method they measured and reported the outcomes: knowledge, attitude, and combined knowledge and attitude.

There were three studies that had assessed the knowledge of the participants; one of these studies investigated *Behvarz*es [21] and the other two studies assessed the general population [27,32]. One of the latter two studies reported quantitative data and the other reported qualitative data, therefore they could not be pooled using meta-analysis. However, all of the three studies showed significant increases in the knowledge of the studied population after the training (table 2). The duration between the pre and post test was two years in one of the studies [32] and not clearly determined in the other two studies (Tables 2 and 3).

Regarding the attitude, there were five studies one of which was performed on the *Behvarz*es [21] and the remaining four studies evaluated the general population [27,32-34]. Three of the latter four studies presented their results quantitatively, however, with different interval periods between training and evaluation, i.e. two weeks [27], two years [34], and one year [33]. Four of the studies also compared the scores in the population under coverage with a control sample population [27,32-34]. Again, all of the four studies showed improvement in the attitude of the subjects after training, but no significant improvement was observed in the control groups. The lack of improvement in the control groups suggests that the observed change has not been due to a time effect.

**Figure 2:**
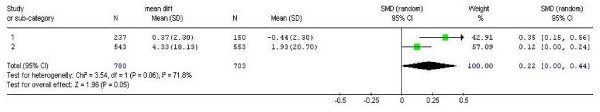
**Meta-analysis of the effect of training on the attitude of general population in the integration program of mental health in PHC**.

In order to calculate the effect size of long term effects of training on the attitude scores a meta-analysis was performed on the latter two studies with one and two year time intervals. A heterogeneity test was performed. The heterogeneity test yielded the following measures: χ^2 ^= 3.54, df = 1, p = 0.06. Considering the significance level of less than 0.1 for possible heterogeneity, a random effects model was used for the meta-analysis. Standardized mean differences of the scores in the two studies were pooled using RevMan Software. Figure 2 shows that the training had an overall significant effect on improving the attitude of the population (Z = 1.96, p = 0.05, effect size = 0.22, 95% CI = 0.0–0.44).

We also found three studies that had reported a combined score for knowledge and attitude of the studied subjects (table 1) [33-35]. All of these studies were conducted on *Behvarz*es, but one of them assessed the effect of training on health technicians (*Karsheas *and *Kardan *are health technicians with four and two years of college education respectively), and *Behdashtyar *(a staff member with a diploma and two years of health education in high school) subgroups, as well [35]. The time interval between training and reevaluation in the studies on *Behvarz*es were one week [33], three months [34] and one year time intervals. Therefore, meta-analysis was not performed on these studies.

## Discussion

This review found some evidence for the efficacy of training on improvement of attitude and knowledge of the health personnel both in short and long term in PHC system. The studies were carried out mainly on *Behvarz*es or general population and very few evidence existed in the other subgroups of subjects. The most robust evidence was found on the change in the attitude of general population, followed by the *Behvarz*es' combined score of attitude and knowledge. Given that making a durable change is one of the main objectives of the integration program, the long term improvement in the knowledge and attitude of the subjects would bear a higher importance. The current meta-analysis showed that the efficacy of training on improvement of the population attitude has lasted for a relatively long period of time. Ignacio et al showed that training could improve knowledge and attitude of health workers equally in all of the six studied countries 18 months after the training [9]. They reported 1.82 and 2.83 improvement in the mean score of attitude and knowledge of the health workers, respectively [9]. Because Ignacio et al did not report the standard deviation of the changes, we could not compare their finding with ours.

Despite the large increase in the number of the studies in the recent two decades in Iran, it seems that health service research has not improved to the same extent [36]. Notably, most of the studies that were reviewed in the current study were different in terms of methods and hence not comparable. For example, definition of variables, assessment tools, or scoring methods that were used in the studies were often not identical. Some of the tools were designed or changed by the researchers themselves, without any validation. This heterogeneity of the methods could at least be partly prevented, if the health governors in each country would provide some standardized assessment tools with a well defined scoring method which researchers could use in their studies on the various aspects of the integration program.

Recently, some electronic databases including IranPsych, IranMedex, and Scientific Information Database have been developed in Iran which provide access to the local studies; most of which are not indexed in the international databases. However, these databases still suffer from several limitations that negatively affect their usefulness. Poorly developed search systems, inability to make queries with complex Boolean search, incomplete coverage, and lack of online access to full text of articles are among these limitations. The current review faced these limitations; however, we tried to have a complete coverage by communicating with the few experts that work in this field.

Improvement in attitude and knowledge of all of the involved groups including the general population has been mentioned as one of the ways to promote the mental health of the community and decrease the need for mental hospitals [37]. Therefore, health planning should specifically address this issue when such a program is designed or revised. Nevertheless, regarding the key role of *Behvarz *in the integration program of Iran, the other subgroups of care providers have largely been neglected in the studies. Therefore, future studies should focus more on the other less studied groups.

## Competing interests

The authors declare that they have no competing interests.

## Authors' contributions

JB, RYN, and ARM designed the study. NM, BG, and SVS performed the searches. NM, BG, and SVS performed the data extraction. JB, RYN, ARM, NM, BG, NA, and SVS performed the analyses and prepared the discussion. All of the authors contributed in preparing the draft of the manuscript and revising it. All of the authors have read and approved the edited manuscript.

## Supplementary Material

Additional file 1 **Search strategy for the electronic bibliographic databases**. The data represent the search strategy used for searching both the international and Iranian electronic bibliographic databases. Click here for file

Additional file 2 **Table S1: The characteristics of the studies evaluating knowledge and attitude of the subjects before and after the training**. The file contains a table describing the characteristics of the studies included in the review. Click here for file
