# Supplementary material for: The change in attitude and knowledge of health care personnel and general population following trainings provided during integration of mental health in Primary Health Care in Iran: a systematic review
Source: Int J Ment Health Syst. 2009 Jun 25;3:15. doi: 10.1186/1752-4458-3-15 (PMC2720378; doi:10.1186/1752-4458-3-15)
Supplement: Additional file 1 — Search strategy for the electronic bibliographic databases. The data represent the search strategy used for searching both the international and Iranian electronic bibliographic databases. [file 1752-4458-3-15-S1.doc]

**Search strategy for the international electronic bibliographic databases:**

1- Search of the articles related to Iran with transcription of the name of different cities: Iran* OR Iran OR Tehran* OR Babol* OR Tabriz* OR Rasht* OR mashhad* OR Mashad* OR Zahedan* OR Fars* OR Shiraz* OR Fasa* OR Ahvaz* OR jundi shapur OR jundishapur OR jondishapour OR jondi shapour OR jundishapour OR jundi shapour OR shahid OR shaheed OR beheshti OR ferdowsi OR Isfahan* OR Esfahan* OR Yasouj* OR Yasuj* OR Arak* OR Qom* OR Kerman* OR Rafsanjan* OR Bakhtaran* OR Urmia* OR Orumieh* OR Oroomieh* OR Oroumieh* OR Behzisti OR Sari* OR Mazandaran* OR Gilan* OR Guilan* OR Guillan* OR Gillan* OR Semnan* OR Yazd* OR Hormozgan* OR Kohgilooye* OR Kohkilooye* OR Kohgilouye* OR Kohkilouye* OR Kohgiluye* OR Kohkiluye* OR Sanandaj* OR baqiyatallah OR baghiatallah OR baghiatollah OR Qazvin* OR azad OR Sabzevar* OR Ardabil* OR Ardebil* OR Bushehr* OR Booshehr* OR Boushehr* OR modares OR modarres OR Ilam* OR Golestan* OR Gorgan* OR Kordestan* OR Kurdistan* OR Kordistsn* OR Kurdestan* OR Artesh OR Karaj* OR Shahrekord* OR Rafsanjan* OR shahed OR Jahrom* OR Shahroud* OR Shahrud* OR Shahrood* OR Kashan* OR Hamedan* OR Hamadan* OR Zanjan* OR Birjand* OR roozbeh OR imam OR emam OR razi OR Tonekabon* OR Tonkabon* OR Lorestan* OR Najafabad* OR army OR Khoramabad* OR Khorramabad* OR Bandar* OR Andimeshk* OR Andymeshk* OR Hashtgerd* OR Khoozestan* OR Khouzestan* OR Sanandaj* OR Shahreza* OR Savojbolag* OR Genaveh* OR Boyerahmad

2- Search of the articles related to mental health: Mental health [mesh] OR Mental Health Services[mesh] OR Community Mental Health Services [mesh] OR Community Mental Health Centers [mesh] OR National Institute of Mental Health [mesh] OR Mental Disorders [mesh] OR Mentally ill Persons [mesh] OR Substance Related Disorders [mesh] OR Environmental Illness [mesh] OR Psychiatric somatic Therapies [mesh] OR Psychological Techniques [mesh] OR Psychiatry [mesh] OR Psychotherapy [mesh] OR Treatment Refusal [mesh] OR Psychophysiologic Disorders [mesh] OR "mental health" OR "psychiatric well being" OR "mental well being" OR mental illness OR psychiatr* OR mental disorder OR mentally ill

3- Search of the articles on attitude and knowledge: "Health Knowledge, Attitudes, Practice"[Mesh] OR attitude$ OR Knowledge OR awareness

4- Combination of the three searches: #1 AND #2 AND #3

**Search strategy for the local electronic bibliographic databases:**

Because of limitations of the local databases in performing Boolean search, we used single words for each search and assessed the results separately. Due to the limited number of the indexed articles the number of retrieved articles in each search was not higher than 100. The following words both in Persian and English were used for the searches:

- Primary health care
- Mental health
- Integration
- Attitude
- Knowledge
- Awareness
