# Supplementary material for: The change in attitude and knowledge of health care personnel and general population following trainings provided during integration of mental health in Primary Health Care in Iran: a systematic review
Source: Int J Ment Health Syst. 2009 Jun 25;3:15. doi: 10.1186/1752-4458-3-15 (PMC2720378; doi:10.1186/1752-4458-3-15)
Supplement: Additional file 2 — Table S1: The characteristics of the studies evaluating knowledge and attitude of the subjects before and after the training. The file contains a table describing the characteristics of the studies included in the review. [file 1752-4458-3-15-S2.doc]

| **Study** | **Source type** | **Studied area** | **Population type** | **Sample** | **Sampling method** | **Sample size** | **Instrument** |
| --- | --- | --- | --- | --- | --- | --- | --- |
| Shahmohammadi, 1990 | Research final report | Shahre Kord | Rural | *Behvarz* and  general population | Random cluster | *Behvarz*: 27 trained and 28 control population: 543 trained and 553 control | Social Attitude Scale, and Test of Knowledge and Attitude of *Behvarz* |
| Bolhari, 1995 | Research final report and article | Savojbolagh | Rural | *Behvarz* and general population | Random cluster | 54 *Behvarz*  387 general population over 15 year | *Behvarz* Attitude Scale, General Population Attitude Scale |
|
| Motallebi, 1996 | Article | Dargaz | Rural | *Behvarz* | Census | 22 *Behvarz* | Researcher made questionnaire |
| Kadivar, 1997 | Research final report | Marvdasht, Kazeron, Sepidan, Darab & FirozAbad | Rural and urban | *Behvarz*, *health technicians* | Random | 295 *Behvarz*, *health technicians* | Questionnaire of Knowledge and Attitude of Health Personnel |
| Bagheri Yazdi, 2001 | Article | Broojen | Rural & urban | General population | Random cluster | Totally 1600 persons over 15 years in both case and control groups & urban and rural | Questionnaire of Knowledge and Social Attitude toward Mental and Neurological Illness, Epilepsy, and Mental Retardation |
| Davasaz, 2004 | Research final report, article, and  dissertation | Andimeshk | Rural | General population | Random cluster | 150 family totally 300 persons in two group | Social Attitude toward Mental Illness Scale |
